# Molecular Identification of the Viruses Associated with Sweetpotato Diseases in Côte d’Ivoire

**DOI:** 10.3390/v17111494

**Published:** 2025-11-12

**Authors:** El Hadj Hussein Tapily, Justin S. Pita, William J.-L. Amoakon, Angela Eni, Kan Modeste Kouassi, Nazaire K. Kouassi, Fidèle Tiendrébéogo

**Affiliations:** 1 Laboratoire d’Innovation pour la Santé des Plantes, UFR Biosciences, Université Félix Houphouët-Boigny (UFHB), Abidjan 22 BP 582, Côte d’Ivoire; 2 The Central and West African Virus Epidemiology (WAVE) for Food Security Program, Pôle Scientifique et d’Innovation, Université Félix Houphouët-Boigny (UFHB), Abidjan 22 BP 582, Côte d’Ivoire

**Keywords:** *Ipomoea batatas*, nationwide survey, viruses, epidemiology, Côte d’Ivoire

## Abstract

Sweetpotato (*Ipomoea batatas*) is a staple crop of strategic importance in West Africa, particularly in Côte d’Ivoire. However, its productivity is increasingly under threat due to viral diseases. Given the lack of updated epidemiological data over the past three decades, a nationwide survey was conducted in September 2023 across 94 fields in 83 locations covering seven agroecological zones of the country. A total of 221 symptomatic and asymptomatic leaf samples were analyzed using PCR for DNA viruses and RT-PCR for RNA viruses. The overall viral incidence rate calculated was 65.61%, with significant regional variations (35–97.18%, *p* < 0.001) and notable differences in the severity of symptoms (*p* = 0.0095). Agroecological zone I was the most affected, while agroecological zones IV and V were the least impacted. Four viruses were identified: cucumber mosaic virus (CMV), sweet potato leaf curl virus (SPLCV), sweet potato feathery mottle virus (SPFMV), and sweet potato chlorotic stunt virus (SPCSV). No badnaviruses were found. CMV was the most common virus found in single infections (43.44%), followed by SPLCV (5.43%). SPFMV and SPCSV were only observed in mixed infections, particularly CMV/SPLCV (14.03%) and CMV/SPFMV (1.81%). Two triple infections were also detected: SPFMV/SPCSV/CMV and SPFMV/SPLCV/CMV. In total, 34 partial coat protein sequences were obtained (28 SPLCV, 4 SPFMV, 1 CMV, 1 SPCSV). Phylogenetic analysis revealed a high similarity between SPLCV isolates characterized in Côte d’Ivoire and those from Burkina Faso, Europe (Spain, Italy), and the Americas (USA, Puerto Rico) with nucleotide identity values ranging from 98% to 100%. The Côte d’Ivoire SPCSV sequence showed 97.92% nucleotide identity with European isolates, whereas SPFMV sequences exhibited greater diversity (77–89% identity) but clustered within the West African lineage. Sweetpotato viral diseases were detected mostly in mixed-cropping fields (66.85%). This work provides the first epidemiological update on sweetpotato viral diseases since 1987 and the first molecular evidence of the nationwide presence of SPLCV and SPCSV in Côte d’Ivoire.

## 1. Introduction

Sweetpotato (*Ipomoea batatas*), a perennial species belonging to the family *Convolvulaceae*, is among the most widely cultivated food crops worldwide. Rich in complex carbohydrates, dietary fiber, β-carotene, and essential micronutrients, it serves as a strategic food resource in many tropical and subtropical regions [1]. In sub-Saharan Africa, this crop not only contributes to food security through its short growth cycle and high yield potential but also supports local economies by providing income for smallholder farmers. Its ability to grow in low-fertility soils and withstand variable climates makes it a valuable crop for enhancing agricultural resilience and promoting environmental sustainability [2,3,4].

In Côte d’Ivoire, sweetpotato cultivation has progressively expanded across various agroecological zones, serving both domestic consumption and the supply of local and regional markets. According to FAO [4], the national production increased from 36,000 tonnes in 1994 to 59,700 tons in 2023, reflecting an upward trend. However, despite its growing importance, sweetpotato production remains hindered by several phytosanitary constraints, particularly viral diseases, which constitute the most significant constraint. These diseases, mainly transmitted by hemipteran vectors such as whiteflies and aphids, can cause yield losses ranging from 50% to 98%, depending on agroecological conditions, varietal susceptibility, and virus interactions [5,6]. The widespread use of vegetative propagation further exacerbates the situation by promoting the accumulation and dissemination of viral pathogens [7,8]. Moreover, mixed infections, often synergistic, intensify symptom expression and reduce both tuber quality and yield [9,10,11].

However, molecular data on virus diversity and phylogenetic relationships remain practically lacking for Côte d’Ivoire isolates. Since the pioneering work of Fauquet and Thouvenel [12], which reported the presence of sweet potato feathery mottle virus (SPFMV) and cucumber mosaic virus (CMV), no identification and molecular characterization based on the sweetpotato viruses have been carried out in the country.

In the context of increasing regional trade, exchange of plant material, and expansion of vector populations, the emergence of new virus strains is highly probable, as already been reported in neighboring West African countries such as Burkina Faso [13] and Benin [14]. Recently, farmers in several regions of Côte d’Ivoire, including Touleupleu, Gonaté, Bouaké, and Oumé, have reported unusual symptoms in their sweetpotato fields, such as leaf curling, yellowing, reduced leaf area, and stunting. These symptoms suggest the possible emergence of novel or recombined sweetpotato viruses in the country.

Therefore, updated molecular investigations are urgently required to identify the viruses currently infecting sweetpotato in Côte d’Ivoire, assess their genetic diversity, and determine their phylogenetic affiliations. This study thus aims to fill this virological gap by identification of the viruses associated with symptomatic and asymptomatic sweetpotato plants throughout the country. The findings will provide essential epidemiological insights to guide the development of effective and sustainable viral disease management strategies for sweetpotato cultivation in Côte d’Ivoire.

## 2. Materials and Methods

### 2.1. Study Sites and Parameters Evaluated

A nationwide field survey was conducted in September 2023. It covered the seven agroecological zones of the country. At each site visited, the exact locality name and geographic coordinates (latitude, longitude, and altitude) were systematically recorded using a handheld GPS. Epidemiological data, incidence, and symptom severity were calculated, and other data (vector abundance, type of cropping system, etc.) were collected using a standardized Kobocollect survey form. In order to avoid redundancy and ensure representative coverage, the sampled fields were spaced approximately 6 km apart.

**Figure 1:**
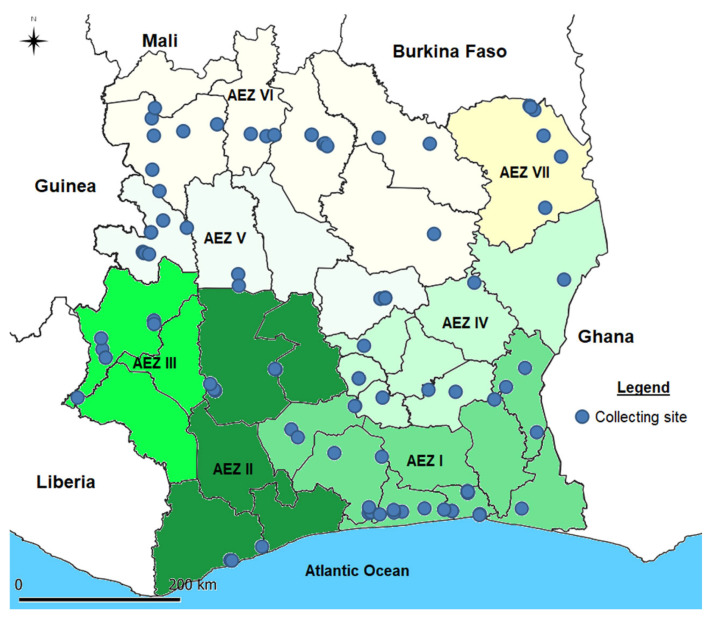
Geographical distribution map of sweetpotato field survey sites in Côte d’Ivoire in September 2023. The different colors represent the limits between the different agroecological zones.

Côte d’Ivoire is divided into seven agroecological zones (ZAEs), which are defined according to climatic and ecological criteria [15] (Figure 1). These zones cover the entire national bioclimatic spectrum: southern humid forest (ZAE I), western humid forest (ZAE II), semi-humid forest (ZAE III), Sudanian savanna (ZAE IV), montane forest (ZAE V), forest-savanna transition zone (ZAE VI), and Guinean savanna (ZAE VII) [16].

Rainfall regimes vary across zones. ZAEs I, II, IV, and V have a bimodal climate, with two rainy seasons alternating with two dry seasons. In contrast, ZAE III experiences a short dry season followed by an extended rainy period. ZAE VI is defined by a prolonged dry season and a shorter rainy season. ZAE VII, meanwhile, displays a sharper alternation between wet and dry periods [17].

### 2.2. Assessment of Incidence, Severity, and Vector Abundance

The incidence of viral disease was calculated as the proportion of symptomatic plants relative to the total number of plants assessed. Epidemiological evaluation including symptom scoring was performed along two diagonal transects within each field, with 15 plants evaluated per transect, giving a total of 30 plants per site [18]. The mean disease incidence (Im) was calculated using the Formula (1). Vector abundance, particularly of whiteflies and aphids, was assessed by counting the number of insects present on the leaves of 30 randomly selected plants along the diagonals of each plot. The leaves were carefully turned over to avoid disturbing or dislodging the insects. In addition, representative whiteflies and aphids were collected using a manual mouth aspirator and preserved in 70% ethanol for subsequent morphological identification. Symptom severity was evaluated following the protocol of McEwan et al. [19] using a 0–9 rating scale (0 = no visible symptoms; 9 = severe symptoms with leaf deformation), as described in Table 1. The mean severity score (Sm) was calculated using the following Formula (2).

Incidence mean (Im) = Number of diseased plants)/Total number of plants assessed(1)

Severity mean (Sm) = ∑Score of diseased plants/Total number of diseased plants(2)

### 2.3. Collection of Sweetpotato Leaf Samples

Fresh leaf samples of sweetpotato, exhibiting a range of symptoms (mild, severe, or asymptomatic), were collected in tubes commonly referred to as 50 mL sterile bottles. The samples were dried with silica gel and stored in a cool, dry place (20–23 °C) until molecular analysis.

### 2.4. Nucleic Acid Extraction and PCR Amplification of DNA Viruses

Total DNA was extracted from leaf samples following the Cetyl Trimethyl Ammonium Bromide (CTAB) method described by Doyle et Doyle [20]. The concentration and purity of the DNA were determined using a NanoDrop (Thermo Fisher Scientific, Waltham, MA, USA) and the DNA working concentration was adjusted to 100 ng/µL. Polymerase chain reaction (PCR) amplifications were carried out in a final reaction volume of 12.5 µL using the GoTaq DNA Polymerase kit (Promega, Madison, WI, USA). Each reaction contained 1× GoTaq buffer, 0.625 U of Taq polymerase, 0.4 µM of each primer, 0.2 mM dNTPs (New England Biolabs, Ipswich, MA, USA), 1 mM MgCl_2_ (Promega), and 100 ng of genomic DNA template.

Two primer sets were used for the detection of DNA viruses: SPG1/SPG2, which target the coat protein gene of sweepoviruses [21], and SPBadna1 3150F/SPBadna2 3550R, which target badnaviruses SPPV-B [22] (Table 2). The PCR cycling conditions were as follows: initial denaturation at 94 °C for 5 min; 30 cycles of denaturation at 94 °C for 40 s, annealing at 60 °C for 40 s, and extension at 72 °C for 1 min; followed by a final extension at 72 °C for 10 min. Amplification products were migrated by electrophoresis on 1% agarose gels at 100 V for 45 min. DNA bands were visualized under UV light using an automated gel documentation system (Axygen Gel Documentation System BL, Union City, CA, USA) following staining with SYBR Safe DNA Stain (Invitrogen, Carlsbad, CA, USA).

### 2.5. Nucleic Acid Extraction and RT-PCR Amplification of RNA Viruses

Total RNA extraction was performed using a modified protocol of the Trizol method described by Tibiri et al. [13]. Trizol was replaced with β-mercaptoethanol as a reducing agent, while lithium chloride (LiCl) was incorporated as a selective precipitant for RNA. This approach yields high-quality RNA extracts that are free from polysaccharide and phenolic contaminants. It is particularly suitable for extracting RNA from tissues that are rich in polyphenols.

Complementary DNA (cDNA) synthesis was conducted using a two-step RT-PCR approach. In the first step, total RNA was heated at 65 °C for 5 min in the presence of dNTPs and oligo d(T) _23_VN primers (New England Biolabs) for SPFMV. The reactions were then rapidly chilled on ice to prevent residual enzymatic activity. In the second step, reverse transcription was carried out at 42 °C for 60 min using M-MuLV reverse transcriptase (Promega) in the presence of an RNase inhibitor, following the procedure described by Prasanth and Hegde [23]. For CMV and SPCSV viruses, random hexamer primers (New England Biolabs) were used, the first step consisting of a 5 min incubation at 25 °C. The second step consisted of reverse transcription performed at 42 °C for 60 min using M-MuLV reverse transcriptase (Promega) in the presence of an RNase inhibitor. An inactivation step of the enzyme at 65 °C for 20 min was performed.

The resulting cDNA was quantified using a NanoDrop spectrophotometer (Thermo Fisher Scientific) and normalized to a concentration of 150 ng/µL. PCR amplification was then performed in a final volume of 12.5 µL containing 1× GoTaq buffer, 0.625 U Taq polymerase, 0.4 µM of each primer, 0.2 mM dNTPs (New England Biolabs), and 1 mM MgCl_2_ (Promega). Specific primer pairs (Table 2) were used for the detection of the viruses targeted. PCR cycling conditions followed the established protocols of Tibiri et al. [13] and Chabi et al. [14].

**Table 2:** PCR primer pairs for detection of sweet potato virus.

Type of Virus	Virus	Primers	Sequences (5′-3′)	Size (pb)	References
DNA Viruses	Sweepovirus (SPLCV)	SPG1	CCCCKGTGCGWRAATCCAT	912	[21]
		SPG2	ATCCVAAYWTYCAGGGAGCTAA		
	Badna SPPV-B	SPBadna1 3150 F	CTACAACTCTCAACCATATGTCCCTC	1050	[22]
		SPBadna2 3550 R	TGGAACCAAGATCAAGGAAGAA	500	
RNA viruses	Potyvirus (SPFMV)	CP1S	AGTGGGAAGGCACCATACATAGC	945	[23]
		CP1A	GCAGAGGATGTCCTATTGCACACC		
	Crinivirus (SPCSV)	CP-F	ATGGCTGATAGCACTAAAGTCGA	774	[24]
		CP-R	TCAACAGTGAAGACCTGTTCCAG		
	Cucumovirus (CMV)	CMV primer 1	GCCGTAAGCTGGATGGACAA	501-subgroupII, 482-487-subgroupI	[25]
		CMV primer 2	TATGATAAGAAGCTTGTTTCGCG		

### 2.6. Statistical Analysis

The data were analyzed using R software version 4.4.1 (R Core Team, 2024). Variations in the incidence of viral diseases, average symptom severity, and whitefly abundance across sampling areas were assessed using generalized linear models (GLM), followed by likelihood ratio tests (chi-square test or Fisher’s exact test in cases of overdispersion). Post hoc comparisons between groups were performed using Tukey’s test to identify significant differences. Data visualization was performed using the ggplot2 package.

### 2.7. Sequencing and Phylogenetic Analysis

PCR amplifications from DNA viruses (SPLCV) and RT-PCR amplification from RNA viruses (SPFMV, SPCSV, CMV) were separately sequenced using the Sanger method on the Genewiz platform (Germany). Raw sequence reads were quality-filtered and subsequently de novo assembled into high-quality contigs using Geneious Prime (version 2025.0.3). Virus identification was performed by comparison against the GenBank database via the BLASTn tool available on the NCBI platform (https://blast.ncbi.nlm.nih.gov/Blast.cgi; accessed on 4 November 2025), following the approach described by [26].

For phylogenetic analyses, representative reference sequences from diverse geographic regions were retrieved from GenBank [27]. Multiple sequence alignments were generated using ClustalW with default parameters. Phylogenetic relationships were inferred using the Maximum Likelihood method: the Tamura-Nei model [28] was applied for DNA viruses, whereas the GTR+G+I model (General Time Reversible with gamma distribution and proportion of invariant sites) was used for RNA viruses. Node support was assessed through 1000 bootstrap replicates. All phylogenetic trees were constructed, visualized, and edited using MEGA version 11.

## 3. Results

### 3.1. Geographical Distribution of the Sweetpotato Fields Surveyed

In total, 94 fields were assessed, and the spatial distribution of survey sites is shown in Figure 1. It covered 83 localities spread across 24 administrative regions belonging to seven agroecological zones of the national territory.

Côte d’Ivoire is divided into seven agroecological zones (ZAEs) defined by climatic and ecological criteria. These zones encompass the national bioclimatic gradient, from the southern humid forests (ZAE I) to the Guinean savanna (ZAE VII). In total, 94 sweetpotato fields were surveyed across all seven ZAEs, ensuring national coverage. Higher sampling densities were recorded in the southern and central zones (ZAEs I, V and VI), whereas fewer sites were visited in the northern and western zones (ZAEs II, III and VII). This distribution reflects the major sweetpotato production areas and provides a representative coverage of the diverse agroecological contexts across Côte d’Ivoire.

### 3.2. Symptoms Observed in the Fields

**Figure 2:**
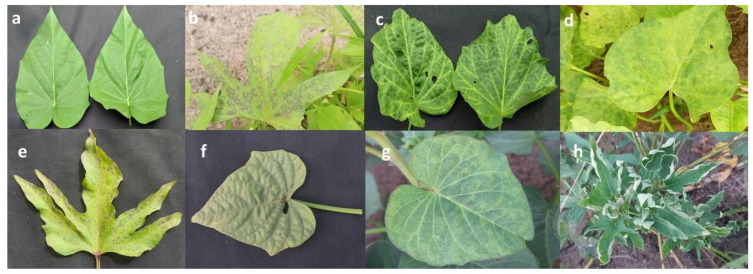
Different types of viral symptoms observed on sweetpotato leaves. (**a**) asymptomatic leaves; (**b**) chlorotic spots; (**c**) vein chlorosis; (**d**) general chlorosis; (**e**) purple spots; (**f**) mottling; (**g**) mosaic and (**h**) leaf curl.

During the survey, both asymptomatic leaves (a = 1) and various virus-like symptoms were observed on sweetpotato leaves across Côte d’Ivoire. The most common symptoms included chlorotic spots (b = 7), vein chlorosis (c = 7), general chlorosis (d = 8), purple spots (e = 6), mottling (f = 8), mosaic (g = 7), and leaf curl (h = 9) (Figure 2).

### 3.3. Disease Incidence, Symptom Severity, and Vector Abundance

**Figure 3:**
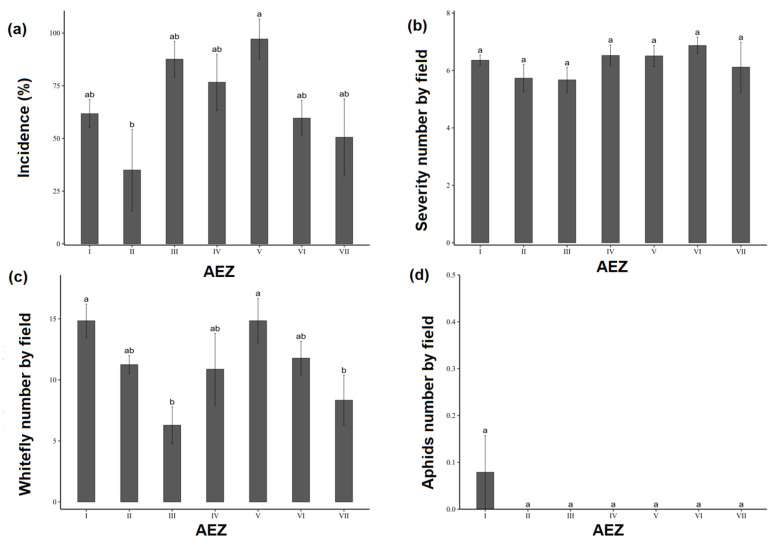
Sweetpotato viral diseases’ epidemiological parameters assessed across seven agroecological zones (AEZ) in Côte d’Ivoire. (**a**) Mean incidence (%) per AEZ; (**b**) Mean severity of symptoms per AEZ; (**c**) Average number of whiteflies per AEZ; (**d**) Average number of aphids per AEZ. Error bars represent standard error (±SE). Columns sharing the same letter are not significantly different according to ANOVA followed by post hoc tests (*p* > 0.05).

**Figure 4:**
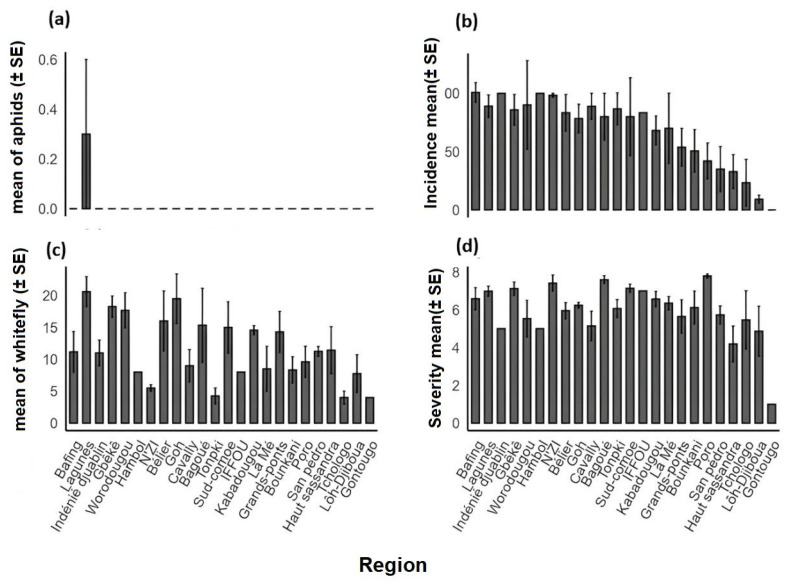
Sweetpotato viral diseases’ epidemiological parameters assessed across regions in Côte d’Ivoire. (**a**) Mean aphid abundance per region (±SE) (**b**) Mean viral disease incidence per region (± SE). (**c**) Mean whitefly abundance per region (±SE). (**d**) Mean symptom severity score per region (±SE). Bars represent mean values; error bars indicate standard error.

**Figure 5:**
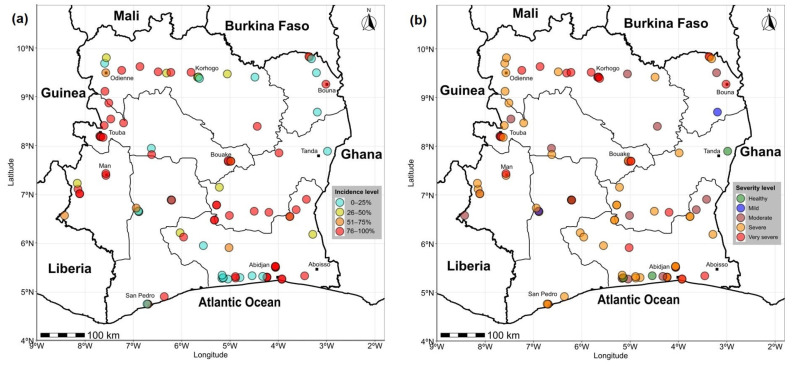
Distribution of viral incidence (**a**) and symptom severity (**b**) in Sweetpotato fields across agroecological zones of Côte d’Ivoire.

The disease incidence (sweetpotato viral infections) showed statistically significant variation among agroecological zones (AEZs) (*p* < 0.05) (Figure 3a). The highest incidence was recorded in AEZ V (97.18 ± 9.52%), followed by AEZ III (85.33 ± 17.67%) and AEZ IV (84.90 ± 21.53%). In contrast, AEZ II displayed the lowest mean incidence (35.00 ± 19.27%). At the regional scale, highly significant differences were observed (*p* < 0.001) (Figure 4). The Bafing, Hambol, Indénié-Djuablin, and N’Zi regions exhibited extremely high incidences, exceeding 90%. On the contrary, Lôh-Djiboua and Gontougo were characterized by particularly low values, below 10%. Intermediate incidence levels (50–75%) were found in Tonkpi, Gbêkê, Lagunes, and Worodougou. The distribution pattern further revealed that high-incidence categories (76–100%) were mainly concentrated in AEZs V, III, and partly in AEZs I, IV, and VI. In contrast, AEZs II and VII included several localities with low incidence, mostly within the 0–25% and 26–50% categories (Figure 5a). Unlike incidence, the mean severity of symptoms did not differ significantly among AEZs (*p* > 0.05) (Figure 3b). Severity scores remained relatively homogeneous, ranging from 5.73 ± 0.47 in AEZ III to 6.87 ± 0.29 in AEZ V. At the regional level, however, severity showed significant differences (*p* = 0.0095). The highest severity levels (6.0–7.0) were observed in Bafing, Bagoué, Gbêkê, Lagunes, N’Zi, and Poro (Figure 4b). Conversely, lower severity values were recorded in Gontougo (1.00 ± 0.00), Haut-Sassandra (4.19 ± 0.94), and Lôh-Djiboua (4.87 ± 1.32). Figure 5b illustrates the distribution of mean severity scores across Côte d’Ivoire. The abundance of insect vectors, particularly whiteflies, showed significant variation among agroecological zones (*p* < 0.05) (Figure 3c). The highest whitefly abundances were recorded in AEZ I and AEZ V, with averages of 14.84 ± 1.37 and 14.84 ± 1.84 individuals per field, respectively. In contrast, aphids (*Aphis* spp.) were not observed, across all agroecological zones (Figure 3d) and regions, except in the Bafing region, where a low mean density of 0.27 ± 0.24 was observed (Figure 4d).

### 3.4. Molecular Detection and Distribution of Sweetpotato Viruses in Côte d’Ivoire

Table 3 summarizes the prevalence of viral infections in the seven agroecological zones (AEZs). A total of 221 samples were analyzed. Among these, 76 samples (34.39%), including 31 symptomatics tested negative for all viruses using the primers employed, while 145 samples (65.61%) tested positive for at least one virus. Single infections (CMV, SPLCV, SPFMV, SPCSV) were abundant, accounting for 48.87% of all sampled plants and 74.48% of all infected plants. Double infections (SPFMV + CMV, SPLCV + CMV) involving two viruses accounted for 15.84% of total samples, while triple infections were rare, occurring in only 1.37% of cases. The most common triple infections involved the SPFMV + SPCSV + CMV and SPLCV + SPFMV + CMV complexes.

**Figure 6:**
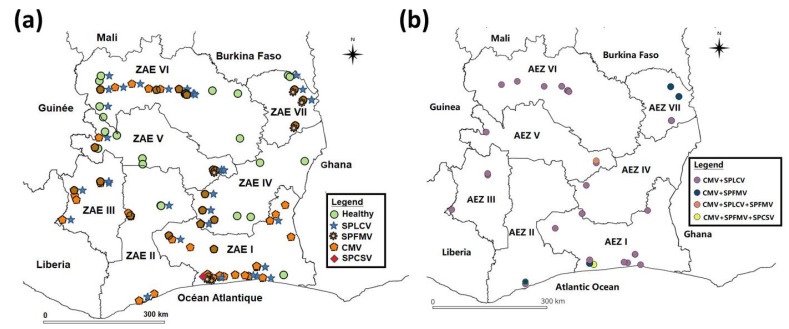
Distribution of viruses infecting sweetpotato in Côte d’Ivoire: (**a**) healthy sample and single infection viruses; (**b**) mixed infections.

Among the pathogens identified, CMV was the most prevalent. It was detected in 43.44% (96/221) of samples in single infection, and in 14.03% (31/221) of cases in co-infection with SPLCV (Table 3). In addition, CMV was involved in both cases of triple infection. SPLCV ranked second in terms of frequency, with an incidence of 5.43% (12/221) in single infections. However, neither SPFMV nor SPCSV was detected in single infection. The latter were only observed in the viral complexes: SPFMV/CMV (1.81%), SPFMV + SPCSV + CMV (0.45%) and SPLCV + SPFMV + CMV (0.45%). Finally, it should be noted that no sweet potato badnavirus (sweet potato badnavirus B, SPPV-B) was detected in any of the samples analyzed. Figure 6a shows the geographic distribution of all individually identified viruses (SPLCV, SPCSV, SPFMV, and CMV), and Figure 6b shows the distribution of mixed infections. It is important to note that this study is the first report of SPLCV and SPCSV in Côte d’Ivoire.

### 3.5. Viral Infection Rates by Cropping System

In this study, the cropping systems observed were mainly intercropping of various crops such as: tomatoes (*Solanum lycopersicum* L.), chillies (*Capsicum* spp.), eggplants (*Solanum melongena* L.), cucumbers (*Cucumis sativus* L.), jute mallow (*Corchorus olitorius* L.), okra (*Abelmoschus esculentus* [L.] Moench), yams (*Dioscorea* spp.) and cassava (*Manihot esculenta Crantz*).

Analysis of viral infection rates according to the cropping systems reveals a significant prevalence of infections in intercropped plots (Table 4). Out of the 145 samples that were infected with at least one virus, 133 were infected with CMV, either alone or in co-infection. Among the infected cases, particularly those with single infection, 64 (66.66%) were from mixed cropping systems, while only 32 cases (33.33%) were from monocultures. When involved in double co-infections, CMV was most often associated with SPFMV and SPLCV, with 4 and 31 cases, respectively, observed mainly in mixed cropping systems. This trend was consistent for all viruses identified. At the same time, SPLCV was detected in 44 samples, most often in the form of an SPLCV + CMV complex, representing 19 cases (61.29%) in mixed cultures compared to 12 cases (38.71%) in monoculture. In single infections, it was identified in 7 (58.33%) samples in mixed culture and 5 (41.66%) samples in monoculture, and was also present in some three-virus complexes, notably SPLCV + SPFMV + CMV. Triple infections, although rare, were limited to two cases: SPLCV + SPFMV + CMV and SPFMV + SPCSV + CMV. SPFMV and SPCSV were detected exclusively in mixed crops. SPFMV was observed in six samples, including four in association with CMV, one in the SPFMV + SPCSV + CMV complex, and one in the SPLCV + SPFMV + CMV complex, while SPCSV was identified in only one sample, in co-infection, notably in the SPFMV + SPCSV + CMV complex. According to Spearman’s rank correlation analysis, differential associations were observed between viral occurrence and cropping system (Table 4). A low significant positive correlation was observed between CMV and intercropping (r = 0.170; *p* = 0.01177). Similarly, the SPFMV + CMV co-infection exhibited a low significant positive correlation (r = 0.147; *p* = 0.02878). In contrast, no significant correlations were observed for SPLCV (r = −0.111; *p* = 0.8696) or for the other viral combinations (Table 4).

### 3.6. Sequencing and Phylogenetic Relationships

A total of 34 partial sequences were obtained from RT-PCR and PCR amplification products. Among these, four sequences corresponded to the coat protein (CP) gene of SPFMV, one to SPCSV, one to CMV and 28 to the gene of the AC1/AC2 of SPLCV.

**Figure 7:**
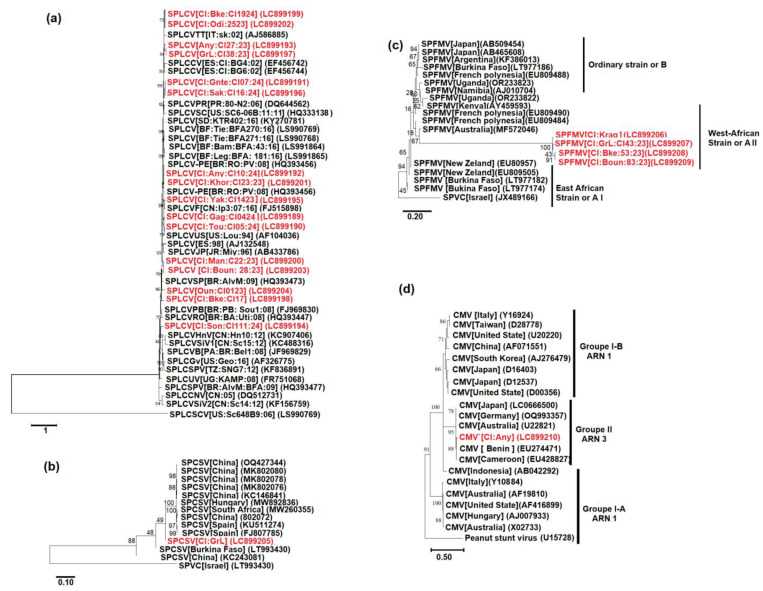
Phylogenetic tree of sweet potato-infecting viruses in Côte d’Ivoire. Phylogenetic tree based on the maximum likelihood method illustrating the relationships among Ivorian isolates of SPLCV (16 isolates), SPCSV (1 isolate), SPFMV (4 isolates), CMV (1 isolate) and various representative isolates of these viruses. (**a**) The tree is based on partial sequences of SPLCV and rooted using the sweet potato leaf curl South Carolina virus (SPLCSCV) (GenBank accession LS990769). (**b**) The tree is based on partial sequences of SPCSV and rooted using sweet potato Virus C (SPVC) (GenBank accession LT993430). (**c**) The tree is based on partial sequences of SPFMV and rooted using SPVC (GenBank accession JX489166). (**d**) The tree is based on partial sequences of CMV and rooted using peanut stunt virus ER RNA1 (GenBank accession U15728). Sequences obtained in the present study are shown in red, while those in black were retrieved from GenBank.

The 28 SPLCV sequences, ranging from 786 nt to 856 nt bp and corresponding to the CP coding region, were successfully amplified using primers SPG1 and SPG2. Sixteen (16) representative sequences have been published to the Genebank database with the following accession numbers (LC899189 to LC899204). BLASTn analysis revealed nucleotide identity values between 98% and 100% with previously described isolates from Italy (Genbank accession number AJ586885), Spain (Genbank accession numbers EF456742; EF45644), Burkina Faso (Genbank accession numbers LS990769; LS991864), the United States (Genbank accession number AF104036), and Puerto Rico (Genbank accession number DQ644562), highlighting a high level of genetic similarity (Figure 7a).

The SPCSV sequence (Genbank accession number LC899205), approximately 774 bp in length, was amplified using primers CP-F and CP-R. BLASTn analysis indicated 97.92% nucleotide identity with two isolates, one from Burkina Faso (LT993430) and another from Spain (Genbank accession number FJ807785). Phylogenetic comparison of the CP gene with 14 reference sequences from different regions confirmed that the Ivorian isolate clustered within the West African strain, grouping with the aforementioned Spanish and Burkinabe isolates (Figure 7b).

The SPFMV sequences (Genbank accession numbers LC899206 to LC899209), ranging from 891 to 897 bp corresponding to the CP coding region, were obtained and sequenced from four samples using primers CP1A and CP1S. The CP sequences of these isolates displayed nucleotide identity levels ranging from 89.39% to 89.92% among themselves. Phylogenetic analysis grouped them within the West African SPFMV group (Figure 7c).

For cucumber mosaic virus (CMV), the obtained sequence (432 bp) with accession number (LC899210) corresponded to the coat protein coding region located on RNA3. BLAST analysis showed 99.14–99.28% nucleotide identity with previously reported isolates from Benin (Genbank accession number EU274471), Cameroon (Genbank accession number EU428827), and Germany (Genbank accession number OP722612.1). In the phylogenetic tree, the Ivorian CMV isolate clustered within subgroup II, together with these reference isolates.

## 4. Discussion

Sweetpotato (*Ipomoea batatas*) is a strategic crop in West Africa, contributing both to food security and to the livelihoods of smallholder farmers [29,30]. However, its productivity is severely constrained by the high prevalence of viral diseases, which can cause yield losses of up to 50–90% under severe mixed infections [31]. Globally, more than 30 viruses have been reported in sweetpotato, among which potyviruses (e.g., SPFMV), criniviruses (SPCSV), cucumoviruses (CMV), and begomoviruses (notably SPLCV) are considered the most damaging [32,33].

In West Africa, and particularly in Côte d’Ivoire, the phytosanitary status of sweetpotato has remained poorly documented over the past decades, despite increasing evidence of emerging viral threats. In response to the recent resurgence of viral infections, often associated with novel symptom expressions, the present study was conducted to fill a critical knowledge gap by providing an updated assessment of viral prevalence and viral genetic diversity in Côte d’Ivoire.

Our results revealed a high and widespread mean viral incidence of 65.6% across surveyed fields, confirming the generally poor quality of planting material used. Although infections were ubiquitous, incidence varied significantly among agroecological zones, ranging from 35.00 ± 19.27% (AEZ II) to 97.18% (AEZ V) (*p* < 0.001). These differences were mainly driven by interactions among cropping practices, vector pressure, and climatic conditions, in agreement with earlier findings by Karyeija et al. [34]. Interestingly, although incidence was high and variable, symptom severity remained moderate and relatively stable across zones (5.73–6.87). This supports previous observations that, in endemic areas, high incidence does not necessarily correlate with severe symptom expression [35]. These results suggest that environmental factors exert limited influence on symptom severity, and point instead to the role of host resistance or attenuated viral aggressiveness. Indeed, resistant cultivars are known to mitigate symptom expression even under high viral pressure [34,36].

In terms of entomology, whiteflies were the most abundant vector species observed at all surveyed fields. The highest densities were recorded in agroecological zones I and V, with respective averages of 14.84 ± 1.37 and 14.84 ± 1.84 individuals per field. Conversely, aphids were virtually absent from all locations, with the exception of the Bafing region, where a very low average density (0.27 ± 0.24 individuals per field) was observed. This finding corroborates reports by Omondi et al. [37] and Thresh [38], which established whitefly as the main vector responsible for SPLCV and SPCSV transmission. By contrast, the low abundance of aphids observed suggests only a marginal epidemiological role, consistent with Valverde et al. [33]. It is important to note that these results are based on a single sampling campaign, and vector populations, particularly aphids, may fluctuate seasonally and from year to year, potentially influencing CMV prevalence.

In this study, agroecological zone III showed the highest viral prevalence (85.33 ± 17.67%) despite the low abundance of whiteflies (*Bemisia tabaci*), the main vector of several sweetpotato viruses, including SPLCV. This observation may reflect multiple factors. First, infections could have occurred prior to vector establishment, meaning that plants were already infected before whitefly populations were evaluated. Second, the use of insecticides in this area may have suppressed vector abundance at the time of sampling without preventing earlier transmission events. Indeed, chemical control reduces adult populations but does not eliminate pre-existing infections or alternative transmission pathways such as the use of infected cuttings for field establishment [11].

Among detected viruses, CMV emerged as the most prevalent, with a molecular detection rate of 43.44%. The results of the sequencing allowed us to confirm the presence of CMV in our samples. CMV was first reported in sweetpotato in Côte d’Ivoire by Fauquet and Thouvenel [12] using ELISA, later confirmed by Kouadio et al. [39] with DAS-ELISA, demonstrating its persistent circulation in Ivorian agroecosystems. More recently, CMV infections in sweetpotato were confirmed in Benin (PCR/seq; [14]) and Ghana (ELISA; [40]), reinforcing its regional significance.

Despite the lower abundance of aphids compared to whiteflies across surveyed fields, the most prevalent virus was the one transmitted by aphids, notably CMV. This observation likely reflects differences in transmission biology and host–vector interactions rather than vector abundance alone. Indeed, aphid-borne viruses are non-persistent and can be transmitted after only a few probing activities, making their spread highly efficient even under low vector densities [41]. Moreover, the vegetative propagation of sweet potato maintains latent infection reservoirs that perpetuate viral circulation regardless of contemporaneous vector pressure [11]. In addition, virus such as CMV can modify plant volatile emissions and surface chemistry, thereby enhancing vector attraction or feeding behavior [42]. Conversely, whitefly-transmitted viruses generally require longer feeding periods and higher population densities to achieve significant transmission [43], which may explain their lower prevalence despite higher vector abundance. Furthermore, temporal shifts between infection onset and vector monitoring, or insecticide applications reducing vector populations post-transmission, could have contributed to the observed patterns [44]. Moreover, importance of CMV lies in its exceptionally broad host range (>1000 plant species), enabling persistence through numerous cultivated and wild reservoirs [45,46]. Furthermore, its non-persistent transmission by more than 80 aphid species enhances its capacity for rapid dissemination within and across cropping systems.

SPFMV was also confirmed in our samples, in line with earlier reports from Côte d’Ivoire [12] and consistent with global data identifying SPFMV as the most widespread sweetpotato virus [34,36,47,48,49,50]. Importantly, this study provides the first report of two emerging viruses in Côte d’Ivoire: SPLCV and SPCSV. SPLCV, detected at an incidence of 5.43%, ranked second after CMV. As a begomovirus, SPLCV is associated with foliar malformation and yield reduction in other regions, underscoring its potential threat. Its limited previous detection likely reflects asymptomatic infections, which are difficult to identify visually, highlighting the value of molecular diagnostics [51]. SPCSV, although detected in only one sample (0.45%), remains particularly concerning, as it is one of the two causal agents of sweetpotato virus disease (SPVD), the most devastating viral disease in sweetpotato, resulting from its synergistic interaction with SPFMV [10,33,52]. Molecular evidence has shown that SPCSV co-infection can increase SPFMV RNA titers more than 600-fold, while SPCSV titers remain stable or decrease compared to single infections [11,31,34]. Thus, even at low prevalence, SPCSV detection should be considered a major warning signal, as its co-circulation with other viruses could have significant impacts on production [13]. Our findings are consistent with Tibiri et al. [13], who also detected SPLCV and SPCSV in Burkina Faso, and Chabi et al. [14] in Benin, confirming their widespread distribution across West Africa.

In our study, PCR targeting sweet potato DNA viruses detected only sweet potato leaf curl virus (SPLCV) in the samples studied. However, a recent study conducted by Name et al. [53] (2025) in Burkina Faso revealed, in addition to SPLCV, the presence of pepper yellow vein Mali virus (PepYVMV), sweet potato leaf curl deltasatellite 3 (SPLCD3), cotton leaf curl Gezira alphasatellite (CLCuGeA), and cotton leaf curl Gezira betasatellite (CLCuGeB). These findings demonstrate that sweet potatoes can be infected by several begomoviruses and satellites (deltasatellite and alphasatellite). The divergence between our conclusions and those of Name et al. [53] could be attributed to the difference in methodologies used. Indeed, Name et al. [53] used rolling circle amplification (RCA) technology coupled with Oxford Nanopore (ONT) sequencing, a method that identifies all circular viruses present in a sample [54,55]. Applying this technique to our own samples could potentially enable us to identify other viruses not identified in this study. Furthermore, this study revealed that 34.39% of the samples tested were negative using the PCR and RT-PCR primers used. This result suggests that the diagnostic approach adopted, which targeted envelope protein genes, has certain limitations. Indeed, genetic variability between viral isolates may hinder primer hybridization, while other viruses not targeted by these primers may have escaped detection. These observations highlight the need to incorporate high-throughput sequencing (HTS) in future studies to enable a more comprehensive and unbiased characterization of the sweet potato virome in Côte d’Ivoire.

This spread is largely attributed to the exchange of infected cuttings for field establishment. The absence of formal seed systems distributing virus-free planting material remains a major aggravating factor, facilitating transboundary dissemination [56].

No badnaviruses were detected in this study, consistent with current knowledge that sweetpotato badnaviruses have only been reported in East and Southern Africa, mainly through small RNA sequencing [57].

Cropping systems significantly influenced viral incidence. In this study, intercropping was associated with a higher incidence (66.85%) compared to monocropping (33.15%), suggesting that heterogeneous cropping environments promote viral persistence and dissemination by providing alternative hosts and favorable niches for vectors such as whitefly and *Aphis* spp. These results align with [34], who demonstrated that host abundance and diversity facilitate interspecific transmission and viral circulation. Conversely, monocropping temporarily limits incidence by reducing intermediate reservoirs, but low genetic diversity increases long-term vulnerability to epidemics. These findings emphasize the need for integrated management strategies combining genetic diversification, resistant varieties, crop rotation, and phytosanitary surveillance to limit both viral spread and monoculture-related risks. Correlation analysis further supports these trends. Significant positive associations were detected between intercropping and both cucumber mosaic virus (CMV; r = 0.170, *p* = 0.01177) and sweet potato feathery mottle virus (SPFMV) + CMV co-infection (r = 0.147, *p* = 0.02878). These results indicate that mixed cropping systems may enhance the dissemination of certain viruses, particularly those vectored by aphids such as *Aphis gossypii*, the principal transmitter of CMV [31,58]. Increased plant diversity within intercropping systems likely provides more abundant feeding and reproduction sites for these vectors, thereby facilitating virus circulation within the crop mosaic.

In contrast, Sweet potato leaf curl virus (SPLCV), transmitted by *Bemisia tabaci*, showed no significant relationship with cropping system (r = −0.111, *p* = 0.8696). This suggests that its spread depends primarily on the use of infected planting material and vector mobility rather than on cropping configuration [36]. Similarly, the absence of correlation for complex co-infections (e.g., SPLCV + SPFMV + CMV or SPFMV + SPCSV + CMV) likely reflects both their low detection frequency and independent transmission dynamics. Overall, the low correlation coefficients (|r| < 0.2) imply that although interactions between cropping systems and viral presence exist, cultivation mode alone is not the main determinant of viral distribution. Rather, factors such as the exchange of vegetative cuttings, vector abundance, and local environmental conditions exert a stronger influence on sweet potato viral epidemiology [12]. Phylogenetic analysis provided insights into the evolutionary dynamics of sweetpotato viruses. SPLCV (*n* = 28) and SPCSV (*n* = 1) isolates were highly homogeneous (>98% nucleotide identity), clustering with strains from Burkina Faso, Europe, and the Americas. This homogeneity suggests either recent introductions via planting material exchanges or active regional circulation, as proposed by Sseruwagi et al. [18] and Tibiri et al. [13]. However, this interpretation remains tentative without complete genome sequences, underscoring the need for next-generation sequencing (NGS) to refine genetic structure and evolutionary trajectories.

In contrast, SPFMV isolates (*n* = 4) exhibited unexpected diversity (89.39% to 89.92% % identity), including a distinctive clade specific to Côte d’Ivoire. In contrast, the four SPFMV isolates displayed unexpected genetic diversity and formed a clade unique to Côte d’Ivoire, likely reflecting local diversification within the country’s agroecosystems. However, as only partial coat protein sequences were analyzed, full-genome studies are required to determine whether this pattern results from adaptive evolution or neutral genetic drift. This observation aligns with Tumwegamire et al. [59], who reported significant regional genotypic variability in SPFMV. The emergence of an Ivorian clade underscores the importance of continuous molecular surveillance to anticipate highly divergent strains that may alter infection severity or synergize with other viruses.

## 5. Conclusions

This study provides an assessment of the viruses associated with sweet potato diseases in Côte d’Ivoire, revealing a high overall incidence (65.6%) and clear agroecological differences in disease prevalence. CMV was the most frequently detected virus, confirming its persistent circulation in West African agroecosystems and highlighting its epidemiological significance due to its broad host range and efficient transmission by aphids. The first detection of SPLCV and SPCSV in Côte d’Ivoire represents an important update to the regional phytosanitary context, as both viruses are key components of severe disease complexes such as SPVD. Although SPCSV was rare, its presence underscores the potential for synergistic interactions with SPFMV. Additionally, the identification of a distinct SPFMV clade indicates localized viral diversification within Ivorian agroecosystems, reflecting dynamic evolutionary processes.

Our findings also demonstrate that cropping systems influence viral incidence, with intercropping associated with higher incidence and monoculture with lower incidence. These results underscore the need for integrated management strategies combining the deployment of resistant varieties, the establishment of clean seed systems, and strengthened phytosanitary surveillance.

Finally, the absence of full-genome data for key viruses, particularly CMV, remains a critical knowledge gap. Future studies should prioritize high-throughput sequencing to resolve viral population structures and monitor emerging variants and unidentified viruses for Côte d’Ivoire. Integrating such molecular surveillance into breeding and management programs will be essential to safeguard sweet potato production and food security in Côte d’Ivoire and across West Africa.

## Figures and Tables

**Table 1:** Description of the symptom severity scores.

Score	Description
1	No symptoms
2	Very mild symptoms (mosaic, chlorosis)
3	Symptoms visible on less than 5% of the plant’s leaves
4	Symptoms visible on 6 to 15% of the plant’s leaves
5	Symptoms visible on 16 to 33% of the plant’s leaves (less than 1/3)
6	Symptoms visible on 34 to 66% of the plant’s leaves (less than 2/3)
7	Symptoms visible on 67 to 99% of the plant’s leaves (more than 2/3)
8	Symptoms visible on 100% of the plant’s leaves (no stunting)
9	Symptoms visible on 100% of the plant’s leaves (with stunting or plant death)

**Table 3:** Viral infection across different agroecological zones.

AEZs	Number of Samples	Number of Healthy Plant	Single Infection (%)				Mixed Infections (%)			
			SPLCV	SPFMV	SPCSV	CMV	SPFMV + CMV	SPLCV + CMV	SPFMV + SPCSV + CMV	SPLCV + SPFMV + CMV
I	74 (100%)	10 (13.51%)	2 (2.70%)	0	0	49 (66.22%)	1 (1.35%)	11 (14.87%)	1 (1.35%)	0
II	16 (100%)	2 (12.5%)	0	0	0	11 (68.75%)	1 (6.25%)	2 (12.5%)	0	0
III	20 (100%)	5 (25%)	1 (5%)	0	0	11 (55%)	0	3 (15%)	0	0
IV	19 (100%)	10 (52.63%)	2 (10.52%)	0	0	6 (31.58%)	0	1 (5.26%)	0	0
V	32 (100%)	19 (59.38%)	2 (6.25%)	0	0	6 (18.75%)	0	4 (12.5%)	0	1 (3.12%)
VI	45 (100%)	24 (53.33%)	3 (6.67%)	0	0	9 (20.00%)	0	9 (20.00%)	0	0
VII	15 (100%)	6 (40%)	2 (13.33%)	0	0	4 (26.67%)	2 (13.33%)	1 (6.67%)	0	0
Total	221 (100%)	76 (34.39%)	12 (5.43%)	0	0	96 (43.44%)	4 (1.81%)	31 (14.03%)	1 (0.45%)	1 (0.45%)

Notes: The values expressed as percentages were calculated according to agroecological zones. - SPFMV and SPCSV were detected only in mixed infections, mainly with CMV or SPLCV.

**Table 4:** Distribution and correlation between the presence of sweet potato viruses and the cultivation system (Spearman’s test).

Virus	Samples Found in Monoculture n (%)	Samples Found in Intercropping n (%)	Total	Correlation Coefficient (r)	*p*-Value
CMV	32 (33.33%)	64 (66.66%)	96 (100%)	0.170	0.012 *
SPLCV	5 (41.66%)	7 (58.33%)	12 (100%)	−0.111	0.87
SPFMV + CMV	0 (0.00%)	4 (100%)	4 (100%)	0.147	0.029 *
SPLCV + CMV	12 (38.71%)	19 (61.29%)	31 (100%)	0.0623	0.36
SPFMV + SPCSV + CMV	0 (0.00%)	1 (100%)	1 (100%)	0.0548	0.42
SPLCV + SPFMV + CMV	0 (0.00%)	1 (100%)	1 (100%)	0.0548	0.42
Total	49 (33.79%)	96 (66.21%)	145 (100%)	-	-

* *p*-value < 0.005.

## Data Availability

The raw data supporting the conclusions of this article will be made available by the corresponding author on request.
